# Autophagy Stimulus-Dependent Role of the Small GTPase Ras2 in Peroxisome Degradation

**DOI:** 10.3390/biom10111553

**Published:** 2020-11-14

**Authors:** Fahd Boutouja, Harald W. Platta

**Affiliations:** 1 Biochemie Intrazellulärer Transportprozesse, Ruhr-Universität Bochum, 44801 Bochum, Germany; fahd.boutouja@uni-mainz.de; 2 Institute of Pathobiochemistry, Johannes Gutenberg-University Mainz, 55099 Mainz, Germany

**Keywords:** autophagy, peroxisomes, pexophagy, rapamycin, mTOR, Ras2

## Abstract

The changing accessibility of nutrient resources induces the reprogramming of cellular metabolism in order to adapt the cell to the altered growth conditions. The nutrient-depending signaling depends on the kinases mTOR (mechanistic target of rapamycin), which is mainly activated by nitrogen-resources, and PKA (protein kinase A), which is mainly activated by glucose, as well as both of their associated factors. These systems promote protein synthesis and cell proliferation, while they inhibit degradation of cellular content by unselective bulk autophagy. Much less is known about their role in selective autophagy pathways, which have a more regulated cellular function. Especially, we were interested to analyse the central Ras2-module of the PKA-pathway in the context of peroxisome degradation. Yeast Ras2 is homologous to the mammalian Ras proteins, whose mutant forms are responsible for 33% of human cancers. In the present study, we were able to demonstrate a context-dependent role of Ras2 activity depending on the type of mTOR-inhibition and glucose-sensing situation. When mTOR was inhibited directly via the macrolide rapamycin, peroxisome degradation was still partially suppressed by Ras2, while inactivation of Ras2 resulted in an enhanced degradation of peroxisomes, suggesting a role of Ras2 in the inhibition of peroxisome degradation in glucose-grown cells. In contrast, the inhibition of mTOR by shifting cells from oleate-medium, which lacks glucose, to pexophagy-medium, which contains glucose and is limited in nitrogen, required Ras2-activity for efficient pexophagy, strongly suggesting that the role of Ras2 in glucose sensing-associated signaling is more important in this context than its co-function in mTOR-related autophagy-inhibition.

## 1. Introduction

The growth and division of cells is closely connected to environmental signals, such as the availability of nutrients. Changing accessibility of nutrient resources can induce the dynamic reprogramming of the cellular metabolism in order to adapt the cell to the altered conditions [1]. The regulation of growth and growth-correlated properties of yeast cells in response to nutrient signals mainly depend on two signaling pathway systems, in which the kinases mTOR (mechanistic target of rapamycin) and PKA (protein kinase A) are among the corresponding central factors. Both systems support metabolism, cell growth, and proliferation by activating the gene expression of factors required for ribosome biogenesis, nutrient uptake, and storage. Both pathways can share certain factors or targets, but the exact molecular interplay of mTOR- and PKA-signaling is still under debate. One major difference lies in the activating signals. While the mTOR complex 1 (TORC1) is mainly involved in the sensing of intracellular nitrogen-signals such as amino acids, the PKA system responds mainly to the uptake of glucose [1,2,3,4]. 

Moreover, both pathways have also inhibitory effects, namely the prevention of bulk autophagy, which is a non-selective autophagy pathway that is responsible for the degradation of cytosolic substrates upon mTOR inactivation. The reason for the block of autophagy under nutrient-rich and mTOR/PKA activated conditions is that macromolecules do not have to be mobilized from the cell’s own material [3]. 

Less is known about the effect on the selective autophagy pathways. A recent study demonstrated that the inhibition of mTOR via the macrolide rapamycin resulted in the degradation of peroxisomes in the vacuole of *Saccharomyces cerevisiae* [5], suggesting a similar role for mTOR not only in the degradation of cytosolic proteins but also in the breakdown of organelles in yeast under the corresponding conditions. Because we were interested to analyse the unknown contribution of the PKA-pathway we tested the role of the central PKA-signaling module Ras2 on peroxisome degradation under different autophagy-inducing conditions.

The *S. cerevisiae* proteins Ras1 and Ras2 [6] play an important role in the glucose-sensing of *S. cerevisiae* cells via the stimulation of the adenylate cyclase Cyr1 [7] and therefore have a central function in the adaptation of cellular physiology to changing conditions. They belong to the family of Ras (rat sarcoma) proteins, which consists of lipidated small GTPases that are involved in cellular metabolism linked to cell proliferation and cell viability [8,9,10]. Yeast Ras1 and Ras2 are homologous to the mammalian Ras proteins, whose mutant forms are responsible for 33% of human cancers [11,12,13]. Human H-Ras can complement a *RAS1/RAS2*-deficient *S. cerevisiae* strain, strongly suggesting a conserved functional and mechanistic similarity [6,14]. 

In the present study, we have analyzed Ras2 point mutants that are comparable to some of the transforming Ras mutations found in human tumors [6]. The Ras2(G19V) point mutant, which is also called Ras2*^val19^* or Ras2^ON^, is the constitutive active mutant of Ras2 and mimics in this respect the GTP-bound form of Ras2. In contrast, the Ras2(G22A) mutant, which is also referred to as Ras2*^ala22^* or Ras2^OFF^, is the inactive form, which should create a similar situation as the GDP-bound Ras2 [15]. We have tested their contribution to peroxisome degradation under conditions that were depending on glucose-sensing as well as the inhibition of mTOR either via N-starvation or by rapamycin.

## 2. Materials and Methods

### 2.1. Yeast Strains

The *Saccharomyces cerevisiae* deletion mutants used in this study (*pep4*Δ, *pex5*Δ, *ras2*Δ) are based on the wild-type (WT) strain BY4742 background (*MATα his3Δ1 leu2Δ0 lys2Δ0 ura3Δ0)* [16] and were purchased from EUROSCARF (Frankfurt, Germany). 

### 2.2. Plasmids

The generation and induction of the plasmids Pex11-GFP [17], Ras2(G19V)/Ras2^ON^, and Ras2(G22A)/Ras2^OFF^ [15] have been described in the corresponding publications.

### 2.3. Cell Culture Conditions

Yeast complete (YPD; 1% yeast extract, 2% peptone, 2% glucose, pH 7.4), selective minimal glucose media (SD; 0.3% glucose, 0.5% ammonium sulfate, 0.17% yeast nitrogen base without amino acids, auxotrophic amino acids and nucleoside, pH 6.0). Oleate-containing medium used for the proliferation of peroxisomes (0.5% ammonium sulfate, 0.17% yeast nitrogen base without amino acids, auxotrophic amino acids and nucleoside, 0.05% 20% Tween40, 0.1% oleic acid, pH 6.0) has been described previously [18]. In addition to oleate medium, the oleate plates contained 0.1% yeast extract, 0.5% 20% Tween40 and 2.4% agar. The nitrogen-starvation medium (SD(-N)) contained 2% glucose, 0.17% yeast nitrogen base without amino acids, auxotrophic amino acids, and nucleoside, adjusted to pH 6.0. Rapamycin was purchased from Merck/Sigma-Aldrich (Darmstadt, Germany).

### 2.4. Fluorescence Microscopy

1.5 mL culture were pelleted at 4000 rpm for 5 min at room temperature and resuspended in 100 µL of FM4-64/YPD-medium (1/125 volume of 1 mM FM4-64, purchased from Invitrogen Karlsruhe (T3166). After cultivation for 30 min at 30 °C the cell culture was washed twice with YPD-medium and incubated in 1 mL fresh YPD-medium for 2 h at 30 °C using a rotator and aluminium foil to shield from the light. Before the cells were subjected to microscopy, they were harvested at 4000 rpm for 5 min at room temperature, washed twice with 1xPBS-buffer, and then resuspended in 40 to 60 µL 1× PBS-buffer. 

The analysis of live cells was performed with a Zeiss Axioplan microscope and deconvolved with AxioVision 4.1 software (Zeiss, Jena, Germany) [5,17].

### 2.5. Immunodetection

The monoclonal mouse antibody was raised against GFP (Roche, Mannheim, Germany). After elimination of not bound primary antibody the blots were incubated with goat anti-mouse IRDye^®^ 800 CW as secondary antibodies and visualized with the Odyssey^®^ infrared imaging system (LI-COR Bioscience, Bad Homburg, Germany). The uncropped Western Blots are shown in the Supplementary Figure S1. 

### 2.6. Pexophagy Assay

To analyze pexophagy [17,19,20], yeast strains expressing the peroxisomal membrane protein Pex11 C-terminally fused with GFP were grown in two precultures (20 mL overnight and 50 mL for 8 h, OD_600 nm_ = 0.3) in SD-medium at 30 °C. Peroxisomal proliferation was induced by incubating the cells for 16 h at 30 °C (OD_600 nm_ = 0.5) in 100 mL oleate media. In order to induce pexophagy the cells had to be harvested at 4000 rpm for 5 min at 4 °C and washed two times with 5 mL sterile dH_2_O (5 min, 4000 rpm, 4 °C). Cells were resuspended in 1 mL sterile water and 0.5 mL of cell suspension were transferred to 100 mL nitrogen-starvation-media (SD(-N)). Samples of the starting point (T0 samples, 0.5 mL remaining of cell suspension) were taken immediately, harvested for 5 min at 4000 rpm, and prepared by TCA precipitation. The culture was incubated for 23 h at 30 °C. After 23 h the T23 samples (50 mL) were harvested, washed two times, and then prepared by TCA (trichloroacetic acid)-precipitation (as described in [21]).

### 2.7. Peroxisome Degradation in Constitutive Presence of Glucose Induced by Rapamycin

To monitor peroxisome degradation under unselective bulk autophagy conditions based on [19], yeast cells were grown in three precultures (first preculture in 10 mL, second and third in 20 mL, OD_600 nm_ = 0.1) under selective minimal glucose conditions at 30 °C. In order to initiate bulk autophagy, the third preculture (incubated for 16 h at 30 °C) was washed two times and resuspended in 1 mL sterile dH_2_O. 200 µL cell suspension were transferred to 20 mL SD-medium, which was treated finally with 0.2 µg/mL rapamycin (Sigma-Aldrich) and/or DMSO as a control. 

As a starting point of the autophagic degradation process (t = 0 h) the remaining cell suspensions were immediately harvested at 4000 rpm for 5 min at 4 °C and prepared by TCA precipitation. After 23 h incubation at 30 °C the t = 23 h samples were harvested (5 min, 4000 rpm at 4 °C), washed two times and then subjected to TCA precipitation (as described in [21]).

### 2.8. Statistical Analysis

The intensity of free GFP signals on the Western Blots was calculated by Image Studio Lite, LI-COR Bioscience (*n* = 5). The results are presented as means ± standard deviation (SD). The analysis of variance was performed by the use of *t*-test procedures. Unless indicated otherwise, a *p*-value of *p* < 0.001(***) was considered as significant.

## 3. Results and Discussion

The aim of the study was to analyze the dependency of peroxisome degradation on the combination of different Ras2-activity mutants with distinct modes of mTOR-inhibition. For this purpose, cells from the *ras2*Δ strain were transformed with either Ras2^val19^ [15], which is furthermore called Ras2^ON^, or with Ras2^ala22^ [15], which is furthermore referred to as Ras2^OFF^. 

### 3.1. Ras2^ON^ Suppresses and Ras2^OFF^ Supports Peroxisome Degradation after Rapamycin-Treatment of Glucose-Grown Cells

**Figure 1:**
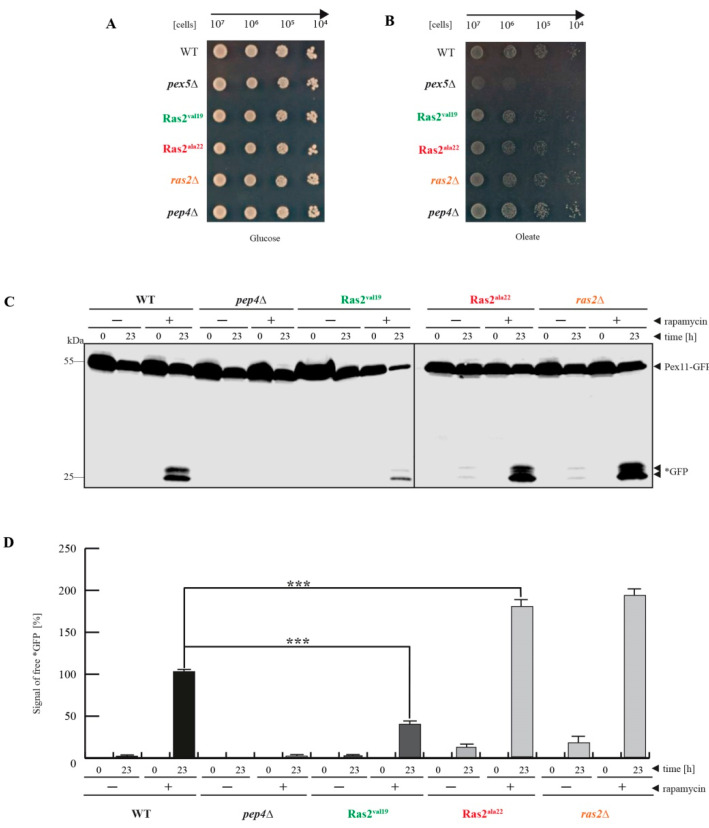
Ras2^ON^ suppresses rapamycin-triggered peroxisome degradation under bulk autophagy conditions. Series of dilutions of cells from wild-type (WT), *pex5*Δ, Ras2^val19^ (Ras2^ON^), Ras2^ala22^ (Ras2^OFF^), *ras2*Δ, and *pep4*Δ were spotted on (**A**) glucose and (**B**) oleate plates. All strains grew well on glucose and only the negative-control *pex5*Δ did not grow on oleate, which excludes pleiotropic growth defects or the inhibition of the biogenesis of functional peroxisomes by the Ras2 versions. (**C**) The influence of the Ras2 activity mutants on peroxisome degradation after inhibition of mTOR with rapamycin in glucose-grown cells was monitored via the stability of the peroxisomal membrane protein Pex11, which was genetically fused to GFP (green fluorescent protein). In WT cells, the Pex11-part of the fusion protein is degraded together with the rest of the organelle, while the GFP-portion is largely stable within then vacuole (*GFP). This process is blocked in *pep4*Δ cells, which served as negative control. The amount of free *GFP is reduced in Ras2^ON^ cells, while it is enhanced in Ras2^OFF^ cells. (**D**) The *GFP signals were measured via densitometry. The statistical analysis of the data (*n* = 5) demonstrates that the amount of generated *GFP is significantly reduced in the Ras2^ON^ cells and significantly elevated in Ras2^OFF^ cells when compared to WT (***, *p* < 0.001). These data strongly indicate that Ras2 supports mTOR in the inhibition of peroxisome degradation in glucose-grown cells.

First, it was ruled out that the Ras2 point mutants could cause a pleiotropic growth defect under the chosen conditions. Defined 10-fold dilutions of the corresponding strains were placed on a glucose plate (Figure 1A). The result showed that all strains grew comparably well on glucose plates, which excludes a general growth defect of the Ras2 mutants. Because peroxisomes are essential for viability of *S. cerevisiae* cells during growth medium lacking glucose and containing oleate as sole carbon source, we also tested the functionality and growth behavior of the corresponding mutants on oleate plates (Figure 1B). While the established negative control of the *pex5*Δ strain did not grow on oleate plates [22], none of the other tested strains displayed a growth inhibition. Even though the Ras2^ON^ mutant grew slightly slower upon close inspection, the cells were still capable of utilizing the oleate from the medium for beta-oxidation, as indicated by the formation of halos around the drop spots where the oleate had been consumed. Therefore, the Ras2 mutants do not affect peroxisome biogenesis. This means that all effects of the Ras2 mutants during peroxisome-degradation assays would be independent of the formation of peroxisomes and only depending on the degradation of peroxisomes.

In order to analyze the impact of the Ras2 mutants on peroxisome degradation, we used glucose-grown cells that were treated with rapamycin in the main-culture for 23 h (Figure 1C). Rapamycin is known to bind to the mTOR-associated FKB12 (FK506-binding protein 12) resulting in the inhibition of mTOR activity, which is followed by the relief of the mTOR-mediated block of bulk autophagy [3]. This enables the unselective degradation of cytosolic cargoes, including peroxisomes [5], to occur. For this experiment, the cells had additionally been transformed with a plasmid encoding the peroxisomal membrane protein Pex11 genetically fused to GFP (green fluorescent protein). The autophagic degradation of peroxisomes can be indicated by the occurrence of free *GFP in the immunoblots because *GFP is relatively stable within the vacuole, while the Pex11-moiety of the fusion protein is degraded together with the rest of the organelle [5,19]. This is the case in the WT cells, where the *GFP signal is only detectable in samples from rapamycin-treated cells after 23 h (Figure 1C). The occurrence of free *GFP is prevented in *pep4*Δ cells that lack the vacuolar master-protease [5]. The data show that the amount of free *GFP is reduced in Ras2^ON^ cells. This indicates that the constitutive active version of Ras2 partially compensates the rapamycin-mediated mTOR inhibition and downregulates peroxisome degradation. In contrast, the samples of the *ras2*Δ strain and the Ras2^OFF^ mutant displayed an elevated amount of *GFP in the +rapamycin/23 h samples. Moreover, already in the −rapamycin/23 h samples of the *ras2*Δ and Ras2^OFF^ strains a certain amount of free *GFP can be detected, while it is absent in the corresponding WT samples. This indicates that also the sole inhibition of Ras2 activity upregulates the autophagic degradation of peroxisomes even in presence of active mTOR, even though the impact is smaller than the mTOR inhibition via rapamycin.

The *GFP signals were measured from five independent experiments via densitometry for quantification (Figure 1D). The statistic results support the conclusion that the amount of free *GFP is significantly reduced in Ras2^ON^ cells compared to WT cells in the +rapamycin/23 h samples. In contrast, the amount is significantly elevated in *ras2*Δ cells and Ras2^OFF^ cells compared to WT in +rapamycin/23 h samples.

In summary, the data indicate that Ras2 has a role in the inhibition of autophagic peroxisome degradation under the analyzed conditions, namely the block of mTOR activity via rapamycin treatment in glucose-grown cells. The activity of Ras2^ON^ can partially compensate the loss of mTOR activity and downregulates peroxisome degradation. Moreover, Ras2 activity seems to contribute constantly to this inhibition in glucose-grown cells, because the simultaneous loss of both Ras2 and mTOR activity enhances the autophagic peroxisome degradation, and the inhibition of Ras2 alone already induced a certain degree of constitutive peroxisome degradation in presence of active mTOR.

### 3.2. Ras2^ON^ Supports and Ras2^OFF^ Suppresses Pexophagy after the Shift of Glucose-Lacking to Glucose-Containing Medium

**Figure 2:**
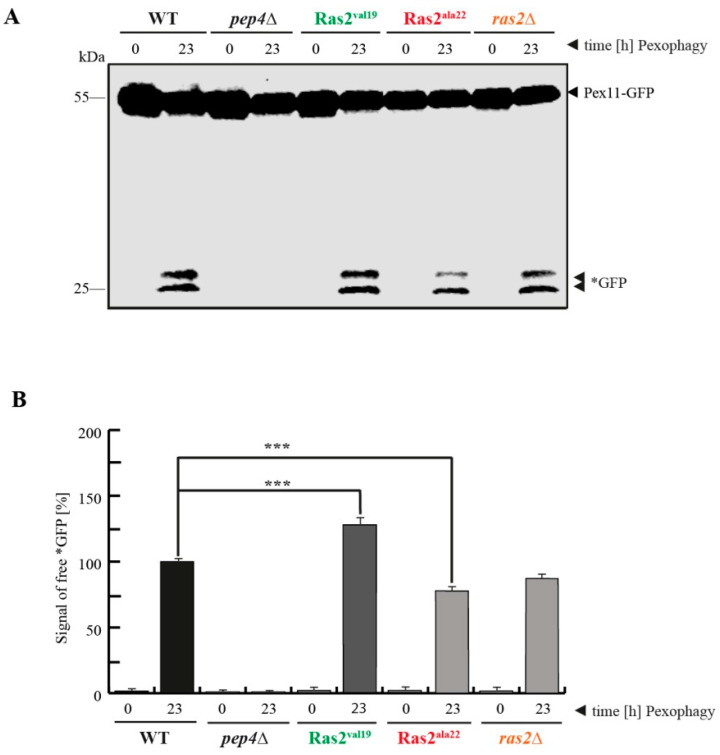
Ras2^ON^ supports pexophagy. (**A**) The impact of the Ras2 activity mutants on pexophagy was analyzed. Pexophagy was induced by shifting cells that had grown on glucose-free oleate medium (where peroxisomes are essential for viability) to a glucose-containing medium (where peroxisomes are not essential) with reduced nitrogen concentration (for the inhibition of mTOR). In wild-type (WT) cells, the Pex11-part of the fusion protein gets degraded in the vacuole with the rest of the peroxisome, while the GFP-portion stays largely stable (*GFP). This process is inhibited in the negative-control *pep4*Δ. The amount of free *GFP is elevated in Ras2^ON^ (Ras2^val19^) cells, while it is reduced in Ras2^OFF^ (Ras2^ala22^) cells. (**B**) The *GFP antibody signals were measured via densitometry. The statistical analysis of the data (*n* = 5) demonstrates that the amount of generated *GFP is significantly higher in the Ras2^ON^ cells and significantly lower in Ras2^OFF^ cells when compared to WT (***, *p* < 0.001). These data strongly indicate that Ras2 is required for efficient pexophagy after the shift from glucose-free oleate medium to glucose-containing pexophagy-medium.

mTOR of glucose-grown cells was inhibited directly by rapamycin in the first part of the study, resulting in an upregulation of autophagy of cytosolic cargoes, including peroxisomes. In the second part, mTOR was inhibited by nitrogen-starvation (Figure 2A), resulting in the selective autophagic degradation pathway of peroxisomes, called pexophagy [23]. The cells were pre-incubated in a glucose-containing medium and then shifted to the first main culture, which completely lacks glucose and contains oleate as the sole carbon source in order to induce peroxisome proliferation (oleate medium). Finally, the cells were shifted to the second main culture, which lacks oleate and contains glucose and a reduced amount of nitrogen (pexophagy medium) in order to induce the pexophagy of the excess of peroxisomes that are not essential anymore. The data show that under these conditions the amount of free *GFP in the sample from the Ras2^ON^ cells (t = 23 h in pexophagy medium) is elevated compared to the WT sample, indicating that Ras2^ON^ is supporting and not interfering with pexophagy. In contrast, the samples from the *ras2*Δ and Ras2^OFF^ cells contained a reduced amount of *GFP. The data suggest that the activity of Ras2 contributes to an efficient breakdown of peroxisomes via pexophagy. 

In order to quantify the data concerning the pexophagy efficiency, the *GFP signals were measured via densitometry (Figure 2B). The statistical analysis revealed that the amount of free *GFP is significantly elevated in Ras2^ON^, while it is significantly lowered in *ras2*Δand Ras2^OFF^ cells.

The data suggest that the most important role of Ras2 under the tested conditions occurs during the shift of the cells from glucose-lacking to glucose-containing medium. The role of Ras2 in glucose-sensing-associated signaling, which had been in general indicated before [2], seems to be more important under these specific conditions than its contribution to the mTOR-mediated block of autophagy in glucose-grown cells, where no shift of glucose-concentration occurred.

The described effects of Ras2 activity on peroxisome breakdown, obtained after the shift of glucose-free to glucose-containing and nitrogen-reduced conditions, are in most parts contrary to those obtained in glucose-grown and rapamycin-induced samples (Figure 1C,D) and therefore strongly indicate a distinct functional role and mode of action of Ras2 under these two conditions.

### 3.3. Time-Dependent Analysis of the Ras2 Activity Effects on Peroxisome Breakdown

The impact of the Ras2 activity mutants on the described different condition-dependent modes of peroxisome degradation was analyzed in a time-dependent manner. 

**Figure 3:**
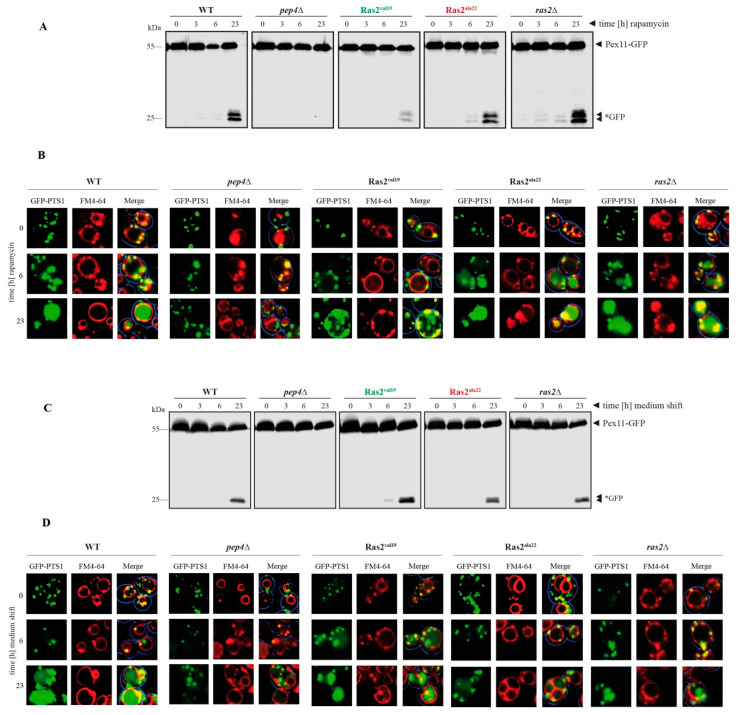
Time-dependent degradation of peroxisomes: rapamycin-triggered peroxisome breakdown occurs fast in Ras2^OFF^ cells, while pexophagy occurs fast in Ras2^ON^ cells. (**A**) The impact of the Ras2 mutants on rapamycin-induced bulk autophagy-related peroxisome degradation was monitored in a time-dependent manner. Compared to WT cells, the Ras2^ON^ (Ras2^va119^) cells exhibited a reduced amount of free *GFP after 23 h. In contrast, Ras2^OFF^ (Ras2^ala22^) showed an elevated level of *GFP, which is already visible after 6 h. (**B**) Peroxisome degradation was monitored via the fluorescence microscope in glucose-grown cells after rapamycin-treatment. In WT, the peroxisomes were detectable as green dots (GFP-PTS1) and the lumen of the FM4-64-stained vacuole showed no signals at t = 0 h. At the end of the rapamycin assay (t = 23 h), the lumen was filled with a diffuse green signal, indicating the breakdown of peroxisomes and the release of free *GFP. This signal was weaker in the vacuoles of Ras2^ON^ cells after 23 h, while it was already visible after 6 h in Ras2^OFF^ cells. (**C**) The influence of the Ras2 mutants on pexophagy was monitored in a time-dependent manner. The cells were grown on oleate medium and then shifted to glucose-containing pexophagy medium. Compared to WT, the Ras2^OFF^ cells exhibited reduced amount of free *GFP after 23 h. In contrast, Ras2^ON^ showed an elevated level of *GFP already after 6 h. (**D**) Pexophagy was monitored via the fluorescence microscope. In WT cells, the peroxisomes were detectable as green puncta (GFP-PTS1) and the lumen of the FM4-64-stained vacuole showed no signals at t = 0 h. At the end of the pexophagy assay (t = 23 h), the lumen was filled with a diffuse green signal, indicating the degradation of peroxisomes and the release of free *GFP. This signal was slightly weaker in the vacuoles of Ras2^OFF^ cells after 23 h, while it was already partially visible after 6 h in Ras2^ON^ cells.

In case of the degradation of peroxisomes under bulk autophagy conditions, glucose-grown cells were treated with rapamycin and samples were taken at the time points 0, 3, 6 and 23 h after the addition (Figure 3A). Again, as also described above (Figure 1C,D), the level of free *GFP at t = 23 h was reduced in Ras2^ON^ cells and elevated in Ras2^OFF^ and *ras2*Δ cells. Moreover, we found that the free *GFP can be already detected after 6 h in the Ras2^OFF^ strain and even after 3 h in the *ras2*Δ cells. This correlation was also verified with fluorescence microscope data (Figure 3B). Peroxisome degradation under bulk autophagy conditions was monitored 0, 6 and 23 h after rapamycin-treatment of glucose-grown cells. Peroxisomes were labeled with the peroxisomal matrix marker GFP-PTS1, while the vacuolar membrane was stained red with FM4-64. In WT cells at t = 0 h, the intact peroxisomes were detectable as green puncta and the lumen of the vacuole showed no signals. At the end of the rapamycin assay (t = 23 h), the vacuolar lumen was filled with a diffuse green signal, indicating the degradation of peroxisomes and the release of free *GFP [19,24]. The intensity of this signal was weaker in the vacuoles of RAS2^ON^ cells after 23 h, while, in contrast, it was already partially visible after 6 h in Ras2^OFF^ and *ras2*Δ cells, therefore indicating a more rapid turnover of peroxisomes in absence of Ras2 activity. 

In the case of the degradation of peroxisomes under pexophagy conditions, cells grown in oleate medium were shifted to pexophagy medium. The samples were collected at the time points 0, 3, 6 and 23 h after the medium shift (Figure 3C). Comparable to the findings described above (Figure 2A,B), the level of free *GFP at t = 23 h was elevated in Ras2^ON^ cells and, correspondingly, reduced in Ras2^OFF^ and *ras2*Δ cells when compared with WT. Additionally, we find that a weak *GFP signal is already detectable after 6 h in the Ras2^ON^ samples, which supports the notion that pexophagy can occur faster with the support of Ras2 activity under the chosen conditions. These results were corroborated with fluorescence microscopy data (Figure 3D). The cells were analyzed 0, 6 and 23 h after the shift from oletate medium to pexophagy medium. While no GFP-PTS1 signals were detectable within the lumen of the vacuole in WT cells at the start of the pexophagy assay (t = 0 h), the diffuse green signal, corresponding to free *GFP, filled the vacuolar lumen at the end of the experiment (t = 23 h). This signal appeared to be slightly weaker in the vacuoles of Ras2^OFF^ and *ras2*Δ cells after 23 h, while it already began to be visible after 6 h in Ras2^ON^ cells.

Therefore, the results show that not only more *GFP can be generated in presence of Ras2^OFF^ (after rapamycin-induction) or Ras2^ON^ (after shift from glucose-deficient to glucose-containing medium), but also that these processes were initiated faster or earlier than in the presence of the corresponding contrary Ras2-activity mutant.

### 3.4. Comparison of the Autophagy Stimulus-Dependent Role of Ras2 Activity Mutants on Different Modes of Peroxisome Breakdown

**Figure 4:**
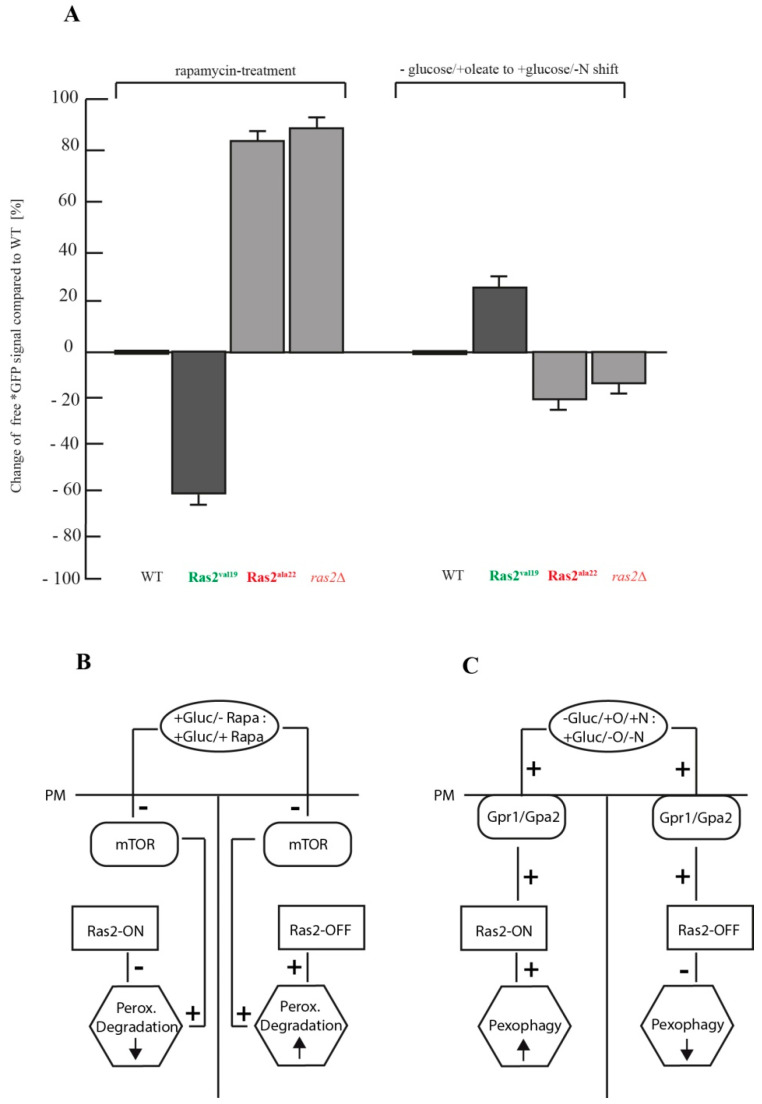
Comparison of the autophagy stimulus-dependent effects of the Ras2 activity mutants on peroxisome degradation. (**A**) The change of the relative amount of generated free *GFP compared to the wild-type (WT) was calculate on basis of the densitometric data of the *GFP signals after 23 h (*n* = 5) after rapamycin-treatment and after the shift from glucose-lacking oleate medium to glucose-containing pexophagy medium. (**B**) Working model part I: Ras2 and mTOR inhibit peroxisome-degradation in glucose-grown cells. After inactivation of mTOR via rapamycin, only the constitutively active Ras2^ON^ (Ras2^val19^) can partially downregulate peroxisome degradation. In case that not only mTOR is inhibited by rapamycin but also the inactivated species Ras2^OFF^ (Ras2^ala22^) is expressed, peroxisome degradation can occur in an enhanced manner. PM = plasma membrane. (**C**) Working model part II: Ras2 is required for efficient pexophagy. Peroxisomes are essential for the cell when grown in oleate as sole carbon source. The shift to glucose-containing medium induced the degradation of an excess of peroxisomes, as they are not essential anymore. This effect is enhanced by the inhibition of mTOR via reduced nitrogen-sources in the medium. Ras2 is known to mediate glucose-sensing induced signaling, involving Gpr1 and Gpa2. Here, Ras2^ON^ supports a rapid sensing of the glucose signal and contributes to enhance pexophagy. In contrast, the Ras2^OFF^ delays the sensing of the presence of the added glucose and therefore decreases pexophagy efficiency.

The effects of the Ras2 activity mutants on the two different modes of peroxisome breakdown were analyzed by comparing the degree of change of the relative amount of free *GFP with the amount detected in the corresponding WT samples after 23 h (Figure 4A). After rapamycin-treatment, Ras2^ON^ showed a 60.7% decreased *GFP level compared to WT, while Ras2^OFF^ displayed an increase of 82.1%. In contrast, after the shift from glucose-lacking oleate medium to glucose-containing pexophagy medium, Ras2^ON^ cells showed an increase of *GFP signals of 28.1%, while Ras2^OFF^ exhibited a decrease of 22.2%.

Based on our data, we propose a working model, which integrates the distinct effects of each Ras2 activity mutant on the context-dependent modes of peroxisome breakdown. The first conclusion is that Ras2 inhibits peroxisome-degradation in cells constantly grown in glucose medium (Figure 4B). We show that after inactivation of mTOR via rapamycin treatment, only the constitutively active Ras2^ON^ can partially downregulate peroxisome degradation. In case that not only mTOR is inhibited but also the inactivated species Ras2^OFF^ is expressed, peroxisome degradation is not limited at all and can occur in an enhanced manner. The second important conclusion is that Ras2 is required for efficient pexophagy (Figure 4C). Peroxisomes are essential for the cell when grown in oleate as sole carbon source. Moreover, oleate induces peroxisomal proliferation [18,22]. The shift to glucose-containing medium resulted in the degradation of an excess of peroxisomes, as they are not essential anymore. This effect is enhanced by the inhibition of mTOR via reduced nitrogen-sources in the medium. Ras2 is known to mediate glucose-sensing induced signaling [2]. Here, Ras2^ON^ should support a rapid sensing of the glucose signal and thereby contributes to enhance pexophagy. In contrast, the Ras2^OFF^ delays the sensing of the presence of the added glucose and therefore slows down pexophagy induction and decreases pexophagy efficiency. Therefore, the overall effect is also smaller when compared to the rapamycin-conditions (Figure 4A), because Ras2 seems to function independently of the mTOR inhibition in this case.

Our novel finding that Ras2 influences the degradation of peroxisomes differently depending on the autophagy stimulus, physiologic situation of the cell, and the mode of mTOR-inhibition is supported by separately published observations. 

In the context of glucose-grown cells, as presented in the first part of our model (Figure 4B), it is interesting to note that the Ras2^ON^ mutant was reported to inhibit the vacuolar proteolysis of the artificial cytosolic cargo Pho8∆60 during unselective bulk autophagy [15]. Pho8∆60 is a cytosolic variant of the vacuolar alkaline phosphatase that lacks the transmembrane domain [19]. Upon shift from standard glucose-containing medium to a glucose medium with reduced nitrogen-sources (SD-N medium), the truncated zymogen Pho8∆60 is transported unselectively with other cytosolic content via autophagosomes to the vacuole during bulk autophagy, where it is activated via limited proteolysis. This transport and activation was drastically reduced in cells with the Ras2^ON^ mutant [15]. In contrast, overexpression of Ras2^OFF^ led to enhanced unselective bulk autophagy of the Pho8∆60 zymogen [15].

In principle, the observed effects of Ras2 activity can be correlated with our finding that Ras2^ON^ can downregulate peroxisome degradation and that the Ras2^OFF^ and *ras2*Δ cells show enhanced peroxisome degradation. However, we inhibited mTOR directly via the addition of rapamycin and not via the N-starvation medium SD-N, as in the mentioned study [15]. Therefore it can be concluded that, even though the final test media were different in the two studies, two criteria were similar: the glucose-conditions were not changed and mTOR was inhibited. In both cases, the cells were still grown on glucose medium and only differed in the mode of mTOR inhibition. The cited study shifted cells from glucose medium with standard N-sources to glucose medium with reduced N-sources (SD-N), while we grew the cells also in a standard glucose medium first before we added rapamycin.

The mechanism by which Ras2 contributes to the inhibition of peroxisomal degradation in glucose-grown cells should be linked to the role of the cAMP/PKA pathway under these conditions. One of the best-known functions of Ras2 is the activation of the adenylate cyclase Cyr1 [7]. The interaction with Ras2 enables Cyr1 to produce an enhanced amount of cAMP, which then activates PKA via an allosteric mechanism [2]. Until now, the cAMP/PKA pathway is only known to contribute to the suppression of unselective bulk autophagy of cytosolic proteins [15]. This function of PKA is executed via the PKA-catalyzed phosphorylation and inactivation of the early autophagy factor Atg13 [25]. This Ras2-signaling mediated phosphorylation of Atg13 by PKA is thought to occur independently of mTOR, which is another Atg13 kinase [25]. It is known that non-phosphorylated Atg13 is required for the autophagic degradation of cytosolic proteins as well as of organelles, such as peroxisomes [26]. Therefore, our finding that Ras2 constitutively downregulates peroxisome degradation in glucose-grown cells is most likely mediated via the cAMP/PKA-axis and the inhibition of Atg13.

The second part of the model (Figure 4C), is based on our analysis of the effect generated by the change from glucose-lacking to glucose-containing medium. This experimental setup induces pexophagy, which has been described as a selective mode of autophagic degradation of peroxisomes [19]. In our assay, we show that Ras2^ON^ can enhance the efficiency of pexophagy, while Ras2^OFF^ and *ras2*Δ reduce the rate of pexophagy. The pexophagy medium is comparable to the SD-N medium, which was used in the above-discussed study on the effect of Ras2 on unselective bulk autophagy of cytosolic proteins [15]. While we used the same Ras2 activity mutants and harvested the cells from the same final growth medium (SD-N/pexophagy medium), the Ras2 activity mutants had contrary effects on the degradation efficiency of the corresponding cargos. The main difference between the two studies lies in the physiologic state of the yeast cells, in which they were before the shift to the glucose/N-starvation medium. In the mentioned study [15], the cells were shifted from glucose full medium to glucose N-starvation medium. In our experiment, the cells were grown on medium that lacked glucose and contained oleate as sole carbon source (oleate medium) prior to the shift to the glucose-containing pexophagy medium. Therefore, the regulation of cellular metabolism during the shift from glucose-free to glucose-containing medium seems to be the dominant task for Ras2 under these conditions. 

Our results on the Ras2 activity mutants concerning pexophagy can mechanistically be best explained by the role of Ras2 in glucose sensing. While a role of Ras2 in pexophagy has not been described before, it has been demonstrated that glucose-sensing in general is required for pexophagy in *S. cerevisiae*, as the deletion of factors involved in glucose-sensing associated signaling, such as the G-protein-coupled receptor Gpr1, its G-protein Gpa2 or the transceptors Snf3 and Rgt2, downregulated pexophagy [27,28]. It is not clear how Ras2 is mechanistically linked to these factors, but it is known that phosphorylated glucose and fructose-1,6-bisphosphate can activate Ras2, possibly via its guanine nucleotide exchange factor Cdc25 [29,30]. GTP-bound Ras2 is thought to be needed for full glucose-sensing signaling activity [2,31], which might also involve cAMP signals, but whose downstream targets concerning pexophagy induction have not been described yet.

A possible role of Ras2 signaling as a link between glucose-sensing and autophagy has been suggested by an observation reported for the vacuole-dependent degradation of the gluconeogenetic enzymes fructose-1,6-bisphosphatase (FBPase) and malate dehydrogenase 2 (MDH2) in *S. cerevisiae* [32]. The WT cells that had been incubated in a medium with a reduced amount of glucose (glucose-starvation) for three days degraded FBPase and MDH2 after the shift to standard glucose medium, because these gluconeogenetic enzymes were not required anymore after the end of glucose-starvation. However, among other tested mutants, the strains *gpr1*Δ, *gpa2*Δ, and *ras2*Δ were not able to break down FBPase and MDH2 [32]. Although the degradation of gluconeogenetic enzymes and peroxisomes is thought to be mediated via different selective autophagy systems, both examples share as a link the Ras2-mediated glucose-sensing signaling. The role and downstream signaling factors and effectors of Ras2 in glucose-sensing signaling have to be different from PKA-linked Atg13-inhibition, as supposed for the glucose-grown cells in part I. In fact, PKA- independent functions of Ras2 have been suggested in the context of the involvement of metabolic-generated reactive oxygen species (ROS) in cellular life span [33]. 

It will be of interest to analyze and compare the two described experimental conditions for peroxisome degradation in more detail in the future. This could include the elucidation of the functional contribution of several Atg proteins during peroxisome degradation under glucose conditions, with a special focus on the pexophagy-receptor Atg36, which is known to be phosphorylated when selective degradation of peroxisomes is induced by N-starvation [34].

It is known that bulk autophagy plays a context-dependent role for the glucose-addicted tumors of mammals [35]. Our yeast study shows that Ras2 up- or downregulates autophagic peroxisome degradation depending on the autophagy-stimulus and physiologic state of the cell.

## 4. Conclusions

The small GTPase Ras2 has an autophagy stimulus-dependent effect on peroxisome degradation.

(1) Ras2 supports mTOR in suppressing peroxisome degradation via bulk autophagy under standard glucose-conditions. After inhibition of mTOR with rapamycin, the Ras2^ON^ version allows only a small rate of peroxisome degradation. The additional inactivation Ras2^OFF^ results in a strongly enhanced peroxisome degradation under glucose conditions.
(2) Ras2 supports glucose-sensing to enhance selective pexophagy. In cells shifted from an oleate-containing/glucose-deficient medium (oleate medium) to a glucose-containing/nitrogen- reduced medium (pexophagy medium), Ras2^ON^ enhances pexophagy, most likely via its central role in glucose-sensing signaling. In contrast, the Ras2^OFF^ displays a reduced rate of pexophagy, most likely because the mutant is unable to fully sense the presence of glucose in the medium and therefore cannot support the breakdown of peroxisomes in presence of glucose.

## Supplementary Materials

The following are available online at https://www.mdpi.com/2218-273X/10/11/1553/s1, Supplementary Figure S1: Uncropped western blots.
