# Supplementary material for: Autophagy Stimulus-Dependent Role of the Small GTPase Ras2 in Peroxisome Degradation
Source: Biomolecules. 2020 Nov 14;10(11):1553. doi: 10.3390/biom10111553 (PMC7696409; doi:10.3390/biom10111553)
Supplement: Supplementary file 1 [file biomolecules-10-01553-s001.pdf]

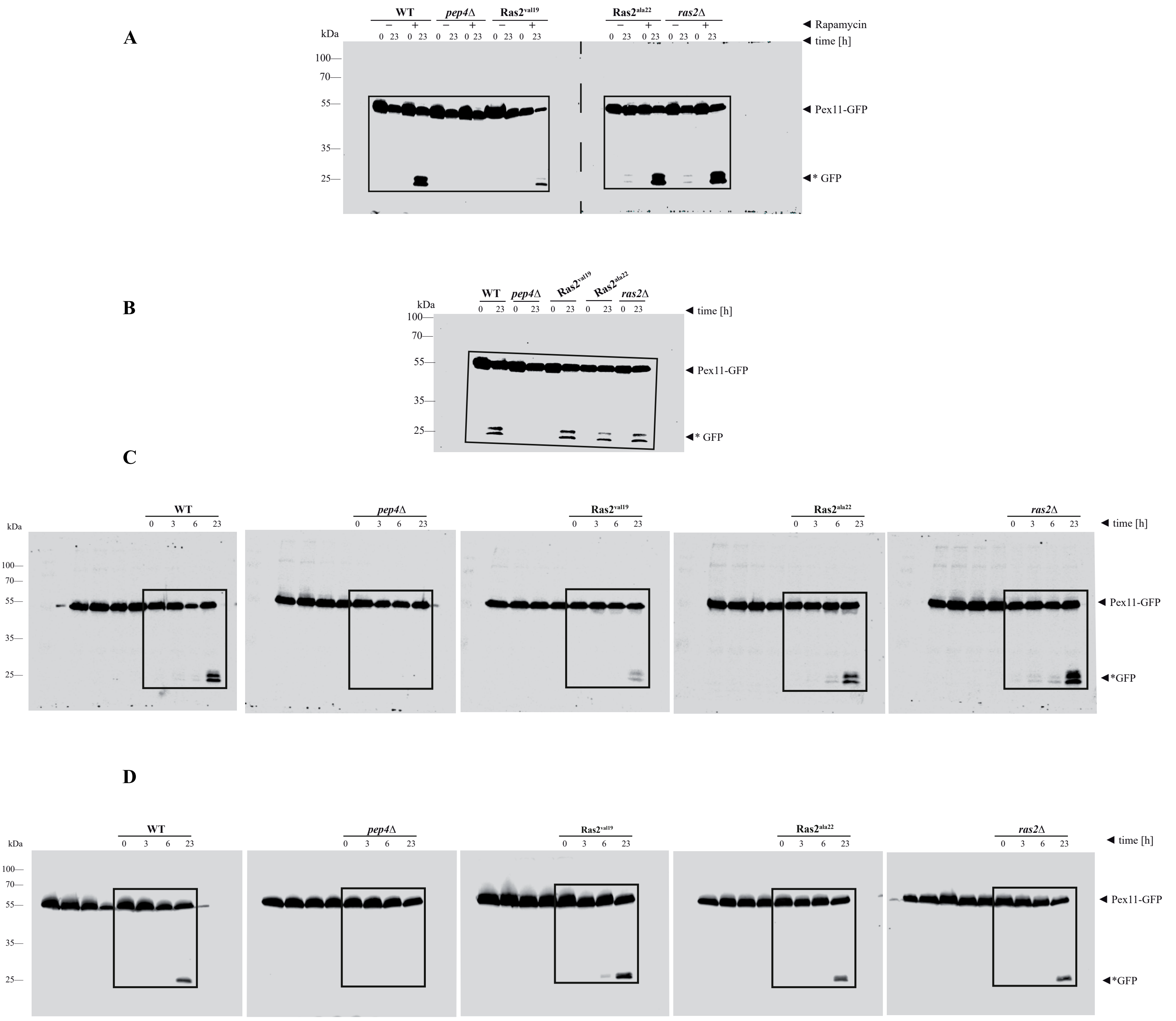

Supplementary Figure S1

(A) Uncropped Western Blots corresponding to Figure 1C (marked area).

(B) Uncropped Western Blot corresponding to Figure 2A (marked area).

(C) Uncropped Western Blots corresponding to Figure 3A (marked area).

(D) Uncropped Western Blots corresponding to Figure 3C (marked area).
